# Differential Requirements of Singleplex and Multiplex Recombineering of Large DNA Constructs

**DOI:** 10.1371/journal.pone.0125533

**Published:** 2015-05-08

**Authors:** Thimma R. Reddy, Emma J. Kelsall, Léna M. S. Fevat, Sarah E. Munson, Shaun M. Cowley

**Affiliations:** 1 Department of Biochemistry, University of Leicester, Leicester, LE1 9HN, United Kingdom; 2 Center for Fisheries, Environment and Aquaculture Sciences, Lowestoft, NR33 0HT, United Kingdom; 3 ES Cell Facility, Center for Core Biotechnology Services, Leicester, LE1 9HN, United Kingdom; Florida International University Bimolecular Sciences Institute, UNITED STATES

## Abstract

Recombineering is an in vivo genetic engineering technique involving homologous recombination mediated by phage recombination proteins. The use of recombineering methodology is not limited by size and sequence constraints and therefore has enabled the streamlined construction of bacterial strains and multi-component plasmids. Recombineering applications commonly utilize singleplex strategies and the parameters are extensively tested. However, singleplex recombineering is not suitable for the modification of several loci in genome recoding and strain engineering exercises, which requires a multiplex recombineering design. Defining the main parameters affecting multiplex efficiency especially the insertion of multiple large genes is necessary to enable efficient large-scale modification of the genome. Here, we have tested different recombineering operational parameters of the lambda phage Red recombination system and compared singleplex and multiplex recombineering of large gene sized DNA cassettes. We have found that optimal multiplex recombination required long homology lengths in excess of 120 bp. However, efficient multiplexing was possible with only 60 bp of homology. Multiplex recombination was more limited by lower amounts of DNA than singleplex recombineering and was greatly enhanced by use of phosphorothioate protection of DNA. Exploring the mechanism of multiplexing revealed that efficient recombination required co-selection of an antibiotic marker and the presence of all three Red proteins. Building on these results, we substantially increased multiplex efficiency using an ExoVII deletion strain. Our findings elucidate key differences between singleplex and multiplex recombineering and provide important clues for further improving multiplex recombination efficiency.

## Introduction

**Fig 1:**
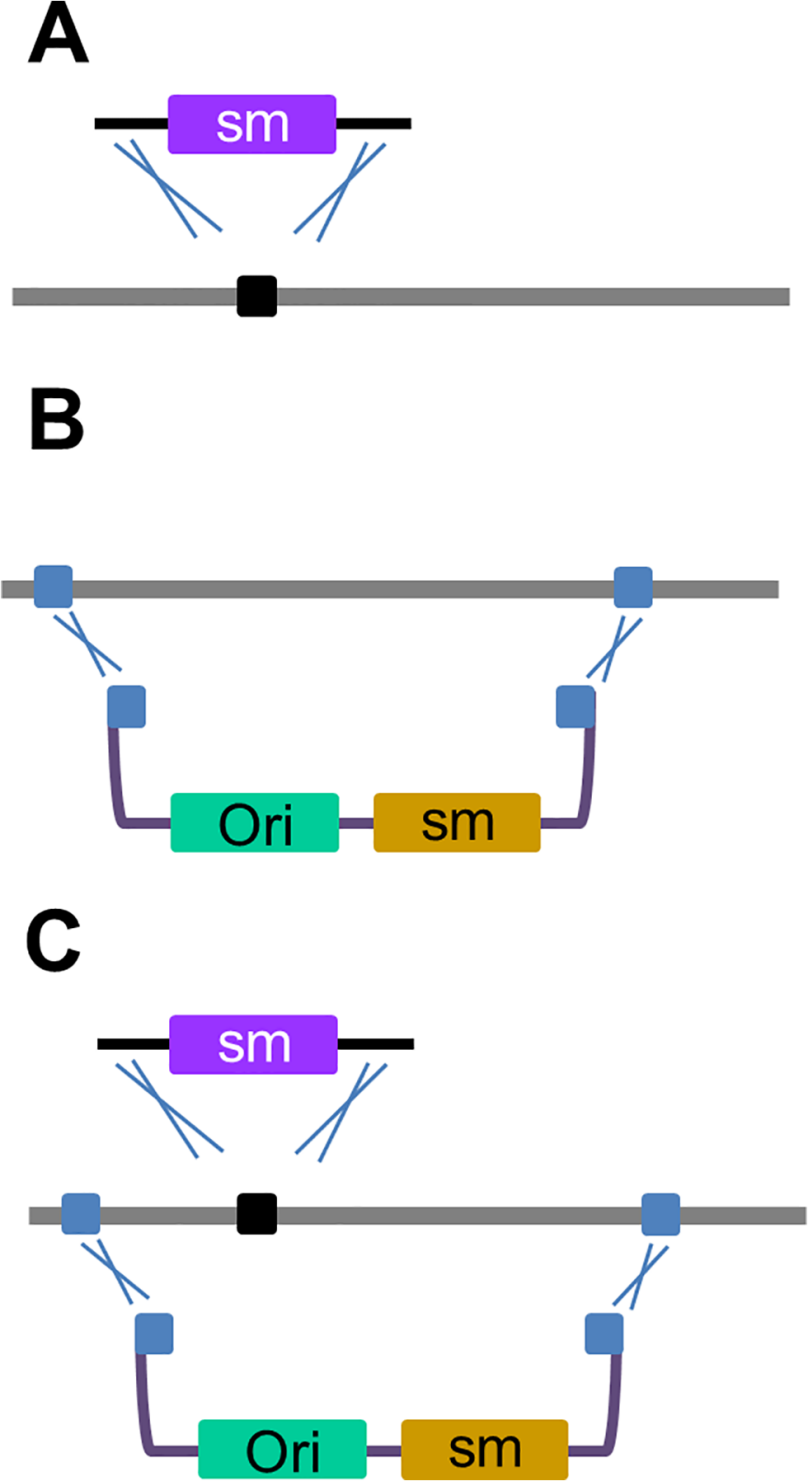
Different types of recombineering processes. (A) Insertional recombination. A selection marker (sm) containing homology to a target site is inserted during the process of DNA replication (dashed line). Gap repair cloning. A gapped plasmid with terminal homology regions to a target site is used to subclone a sequence of interest. (C) Subcloning plus insertion (SPI). Insertion of a cassette occurs simultaneously during subcloning and generates a targeted subcloned plasmid. The template DNA remains unmodified.

A key tool for genetic engineering in bacteria is recombineering, which involves homologous recombination mediated by phage encoded proteins [1,2]. Typical recombineering exercises like the insertion of a gene cassette (Fig 1A) or subcloning of DNA by gap repair (Fig 1B and 1C) require only short regions of homology to the target and generates high recombination efficiencies [3–5]. Consequently, recombineering has enabled the introduction of a variety of genetic modifications including seamless changes [6–8] and has helped greatly accelerate progress in understanding gene function [9–11], isolation of protein complexes [12–14] and exploitation of synthetic metabolites [15–17]. The recombination functions are provided by the Red system of the phage lambda or the equivalent RecET system of the *E*. *coli* cryptic Rac prophage [18,19]. The Red system utilizes three different proteins. Redα is an exonuclease that completely degrades one strand of a double-stranded DNA (dsDNA) and generates a single-stranded DNA (ssDNA) intermediate, which is concomitantly bound by the Redβ single-stranded annealing protein (SSAP) [20–23]. Recombination of the beta coated ssDNA occurs preferentially on the lagging strand of the replication fork and leads to incorporation into newly replicated molecules by a mechanism termed beta recombination [24–26]. Redγ is the third member of the Red system, which inhibits the RecBCD exonuclease and is required for efficient recombination of dsDNA while ssDNA recombination only requires Redβ [27,28].

Standard recombineering practices allow single targets to be modified at a time and can be termed singleplex recombineering. Whilst singleplex recombineering is de rigueur for strain and vector construction applications [29–32], this process is not readily amenable to efficiently creating a more complex set of changes like the introduction of multiple mutations across the genome. To address these limitations, enhanced multiplex recombineering strategies have been recently developed [33–36]. Multiplex recombineering has been used in whole genome recoding [37], the rapid engineering of producer strains [38] and the optimization of metabolite pathways [36]. Thus, overcoming the limitations of singleplex recombineering have opened up exciting avenues to explore new biological functions [39], produce diverse proteins [34,36,40] and to improve biosecurity [41,42]. Multiplexed automated genome engineering (MAGE) is a primary example of a multiplex recombineering technique [34]. MAGE involves the multiplex insertion of oligos at different genomic sites in *E*. *coli* via a cyclical process that generates a population containing a vast combinatorial diversity of mutations. Notably, tagging of the entire translational protein complex for *in vitro* multi-enzyme catalysis (MEC) [43] and the generation of improved lycopene producer strains [34,44] have been achieved with MAGE. Recently, we have described a novel multiplex recombineering methodology using large DNA constructs that permits the simultaneous insertion of whole genes at different genomic targets in the same cell (Reddy *et al*., under review). Double-stranded DNA multiplexed recombineering is a key tool for the construction of novel microbial strains containing complete heterologous metabolic pathways and for the rapid assembly of gene targeting vectors [45]. While the design requirements of MAGE have been well characterized including minimization of oligo secondary structures, mismatch repair evasion and oligo protection [34,46–48], the parameters affecting double-stranded multiplex recombineering need to be clearly defined to elucidate the processes that impact multiplex recombination of large DNA. Here, we have used insertion and gap repair assays to perform a systematic comparison of the requirements of double-stranded multiplex recombineering with singleplex recombineering. We have identified homology length, in vivo template availability and co-selection as the main factors that determine multiplex efficiency. To validate these parameters, we used exonuclease VII deficient strains to increase multiplex recombination efficiency.

## Results

### Longer homology lengths allow efficient multiplexing

**Fig 2:**
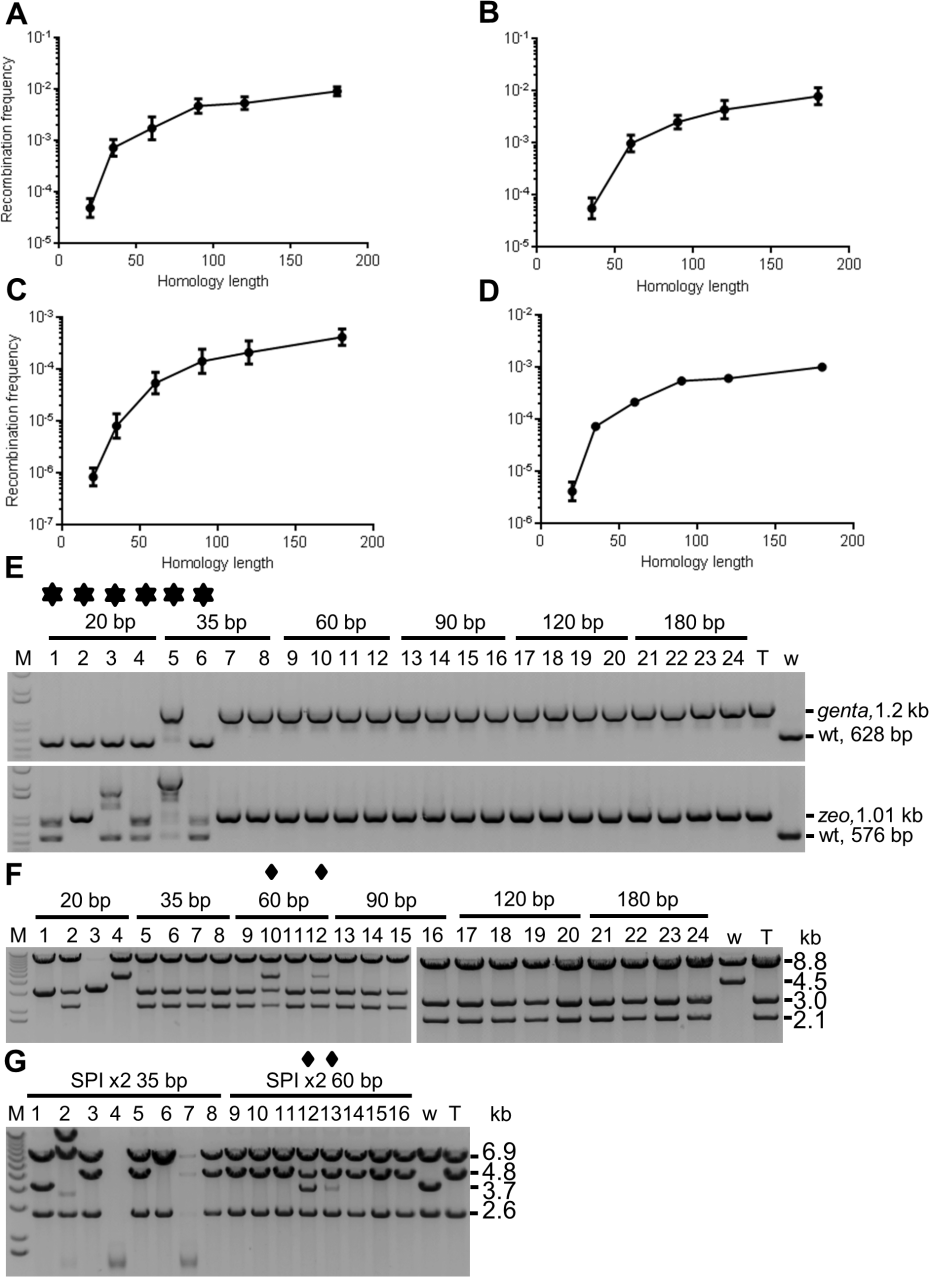
Effect of homology length on singleplex and multiplex recombination. (A) Insertion assay. A Gentamicin lagging strand protected cassette was PCR generated with different homology lengths (20 bp, 35, bp, 60 bp, 90 bp, 120, bp and 180 bp) and inserted at a site of the mouse *P2rx1* gene. Data points represent averages; error bars indicate standard error of mean (*n* = 3). The recombination frequencies plotted here and in the subsequent figures are shown in S1 Table. (B) Gap repair assay. Gap repair was performed at the *P2rx1* locus using p15A *zeo* lagging strand protected subcloning plasmids containing different HLs. Gap repair frequency was calculated using PCR genotyping of 24 clones for each sample (*n* = 3). The 20 bp homology did not yield any correct gap repaired plasmids. (C) Multiplex insertion assay. Homology series of two different Zeocin and Gentamicin lagging strand protected cassettes both containing the same HL were PCR generated and simultaneously inserted at two different sites of the *P2rx1* gene (*n* = 3). (D) SPI assay. A SPI assay was performed at the *P2rx1* gene using a p15A *zeo* lagging strand protected subcloning plasmid containing 230 bp homology regions and the Gentamicin cassettes used in (A) (*n* = 3). (E) PCR analysis of multiplex insertion assay. Recombinants were genotyped with an insertion cassette specific primer and a homology region flanking primer. Positive clones were used to isolate BAC DNA and long range PCR was performed at each of the insertion sites using both homology region flanking primers. The presence of the wild-type (wt) band in a sample indicates the presence of mixtures of BAC plasmids denoted by a star symbol. T, *P2rx1 zeo genta* BAC (positive control); w, *P2rx1* wild-type BAC (negative control); M, 1 kb+ ladder (Invitrogen). (F) Restriction enzyme (RE) analysis of SPI assay with one cassette. Plasmid DNA was digested with EcorV and SspI. Diamond symbol denotes samples containing targeted (T) and non-targeted (w) p15A plasmids. (G) RE analysis of SPI assay with two insertion cassettes. SPI was performed using a p15A *dhfrII* lagging strand protected subcloning plasmid containing 230 bp homology regions and Zeocin and Gentamicin lagging strand protected cassettes containing 35 bp or 60 bp homologies. Clones were analysed with KpnI.

The effect of homology length on recombination frequency was tested using an insertion recombination assay using a Gentamicin cassette containing different lengths of homology (HL) identical to a site of the mouse *P2rx1* gene on a Bacterial Artificial Chromosome (BAC) (S1 Fig). In parallel, a gap repair assay was performed at the same *P2rx1* locus using p15A subcloning plasmids containing a similar set of HLs. Both sets of linear PCR cassettes were asymmetrically modified with two terminal phosphorothioate bonds to protect the strand that could prime DNA synthesis on the lagging strand near the replication fork. The complementary strand contained a 5’ phosphate to promote its degradation by λ exonuclease and to help release the ssDNA recombination intermediate [24]. Efficient insertion required 35 bp of homology and the recombination frequency increased with greater homology to a maximal level observed at 120 bp (Fig 2A). Colony PCR analysis confirmed correct recombination in most samples (data not shown). In contrast, gap repair using 35 bp homology showed the correct subcloned insert in only some of the recombinants (data not shown). However, increasing the homology length to 60 bp showed correct gap repair in most clones and maximal recombination frequency was reached with 120 bp homology similar to singleplex insertion recombination.

Next, the homology requirement of multiplex recombineering was tested. A series of HLs was tested in a multiplex insertion assay using two different antibiotic cassettes both containing the same HL and targeting two different sites of the *P2rx1* gene. Colony counts (Fig 2C) and PCR analysis (Fig 2E) showed that efficient multiplex insertion of both the cassettes on the same BAC DNA required 60 bp of homology. Shorter homologies generated BAC plasmid mixtures. In contrast to singleplex experiments, maximal multiplex insertion was observed with a HL greater than 120 bp. We have previously described a multiplex gap repair assay termed selection for subcloning plus insertion (SPI) that involves the simultaneous insertion of a selection cassette during subcloning [45]. A SPI assay was performed at the *P2rx1* gene using the same p15A subcloning plasmid with long homologies (230 bp HA) and Gentamicin cassettes containing different HLs. SPI assays revealed that whilst correct SPI using one cassette required 35 bp of homology (Fig 2D and 2F), SPI using two cassettes required 60 bp of homology (S2 Fig and Fig 2G), consistent with the requirement of increased homology for multiplex recombination.

To investigate the differential HL requirement of SPI, different HL combinations were of the subcloning plasmid and the insertion cassette tested. Efficient SPI recombination required long homology of both DNA cassettes (S3 Fig). However, SPI recombination was reduced 4 fold when the HL of the subcloning plasmid was shorter than that of the insertion cassette.

### Phosphorothioate protection of DNA is required for efficient multiplex recombination

**Fig 3:**
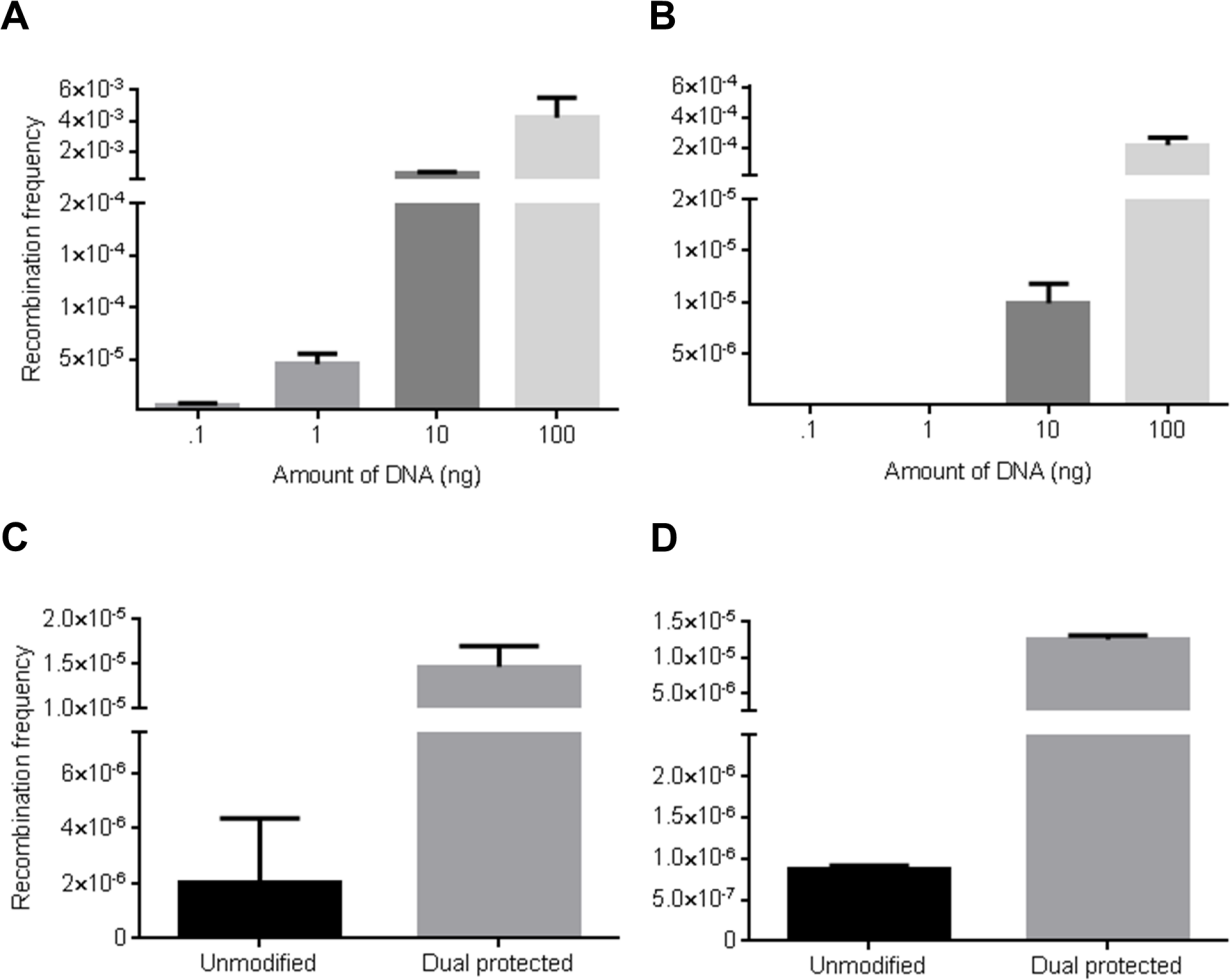
Multiplex recombination is limited by availability of DNA template. (A) Insertion assay. A Gentamicin lagging strand protected cassette was inserted at a site of the *P2rx1* gene. (B) Multiplex insertion. Two different Gentamicin and Zeocin lagging strand protected cassettes were inserted at two different sites of the *P2rx1* gene. Recombination assays were performed with different amounts of each DNA cassette in the electroporation. Values represent averages; error bars indicate standard error of mean (*n* = 4). (C) Multiplex insertion PTO assay. Three different Zeocin, Gentamicin and Blasticidin resistance cassettes were PTO protected or unmodified and inserted at three different sites of the *P2rx1* gene (*n* = 3). (D) SPI PTO assay. SPI was performed using a p15A *dhfrII* subcloning plasmid and the Zeocin and Gentamicin cassettes used in (A) (*n* = 3).

Template availability is likely to be a key factor in multiplex recombineering due to the lower mole ratios of the different cassettes in the total DNA used in electroporation and the difficulty of introducing all the multiple cassettes into the same cell [47]. To investigate the effect of limiting DNA availability, different amounts of the insertion cassettes were tested in singleplex and multiplex insertion assays (Fig 3A and 3B). Multiplex insertion showed a greater decrease in recombination frequency than singleplex insertion with lower amounts of DNA (500 fold lower between 10 ng and 1 ng; compare Fig 3B to 3A), indicating a higher requirement of DNA multiplexing. PCR analysis revealed correct multiplex recombination in all the cloned analysed.

To further substantiate these findings, phosphorothioate modification (PTO) was employed to protect the ends of the linear cassettes and increase the stability of the DNA in vivo. Multiplex assays utilized three different cassettes and compared PTO modification to their unmodified counterparts. As expected, multiplex insertion (> 7 Fold) and SPI (> 14 fold) showed greater recombination with PTO protected cassettes than with the unmodified DNA (Fig 3C and 3D).

### Co-selection enhancement of multiple recombination

**Fig 4:**
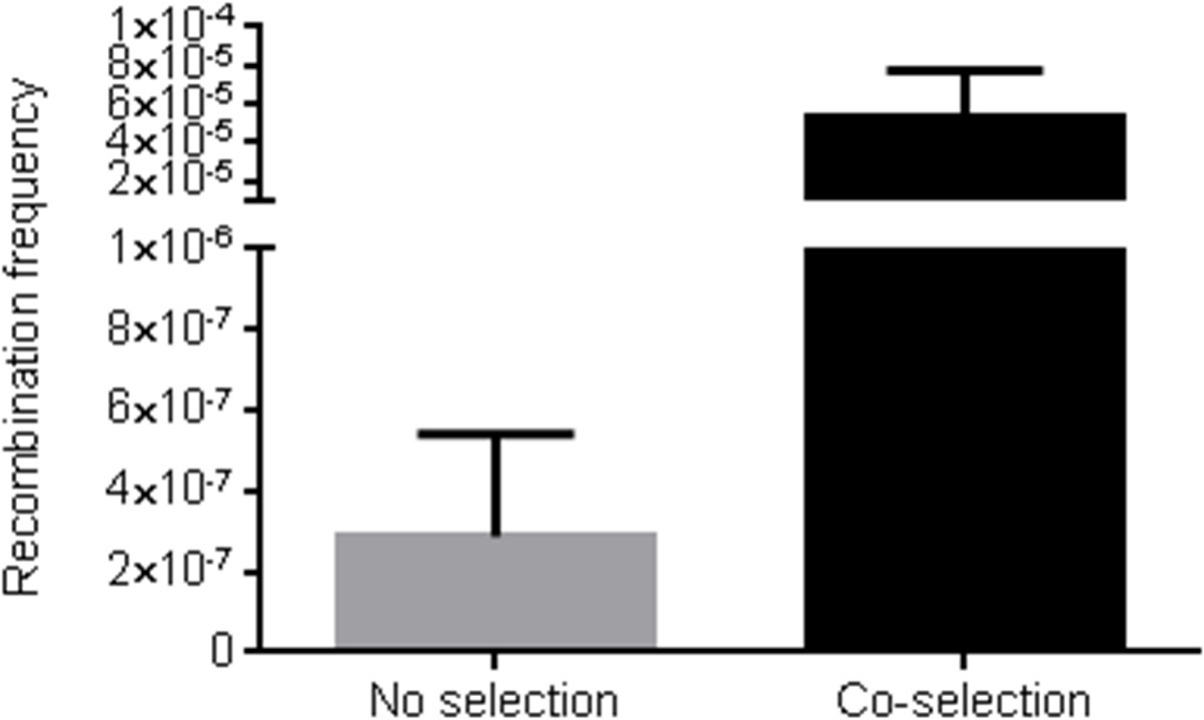
Co-selection is required for efficient multiplex recombination. Multiplex insertion was performed using a pBeloBAC11 plasmid (19.5 kb total size) containing the region of the *P2rx1* gene shown in S1 Fig. The Zeocin and Gentamicin lagging strand protected cassettes were co-transformed with a Kanamycin resistance plasmid and selected with Zeocin and Kanamycin or Kanamycin only. BAC DNA was prepared using the QIAquick Spin Miniprep kit (Qiagen) from each selection scheme and 100 ng of DNA was transformed into HS996 cells and plated with combined Zeocin and Gentamicin selection. Values represent averages; error bars indicate standard error of mean (*n* = 3).

Co-selection has been used in MAGE to increase multiplex allele conversion frequency by simultaneously targeting a selectable marker near the oligo annealing sites [40,49]. Selection allows enrichment of a proportion of cells that are more permissible for DNA uptake and contain an active replication fork near the selection marker. To determine the effect of co-selection in large construct multiplex recombineering, a multiplex insertion assay was performed using two different antibiotic cassettes and either singly selected or not selected. BAC plasmids were prepared, re-transformed and then plated with antibiotic selection for both cassettes. Multiplex insertion of both cassettes was nearly 184 fold higher with single selection than without any selection (Fig 4).

### Requirement of the different Red proteins

**Fig 5:**
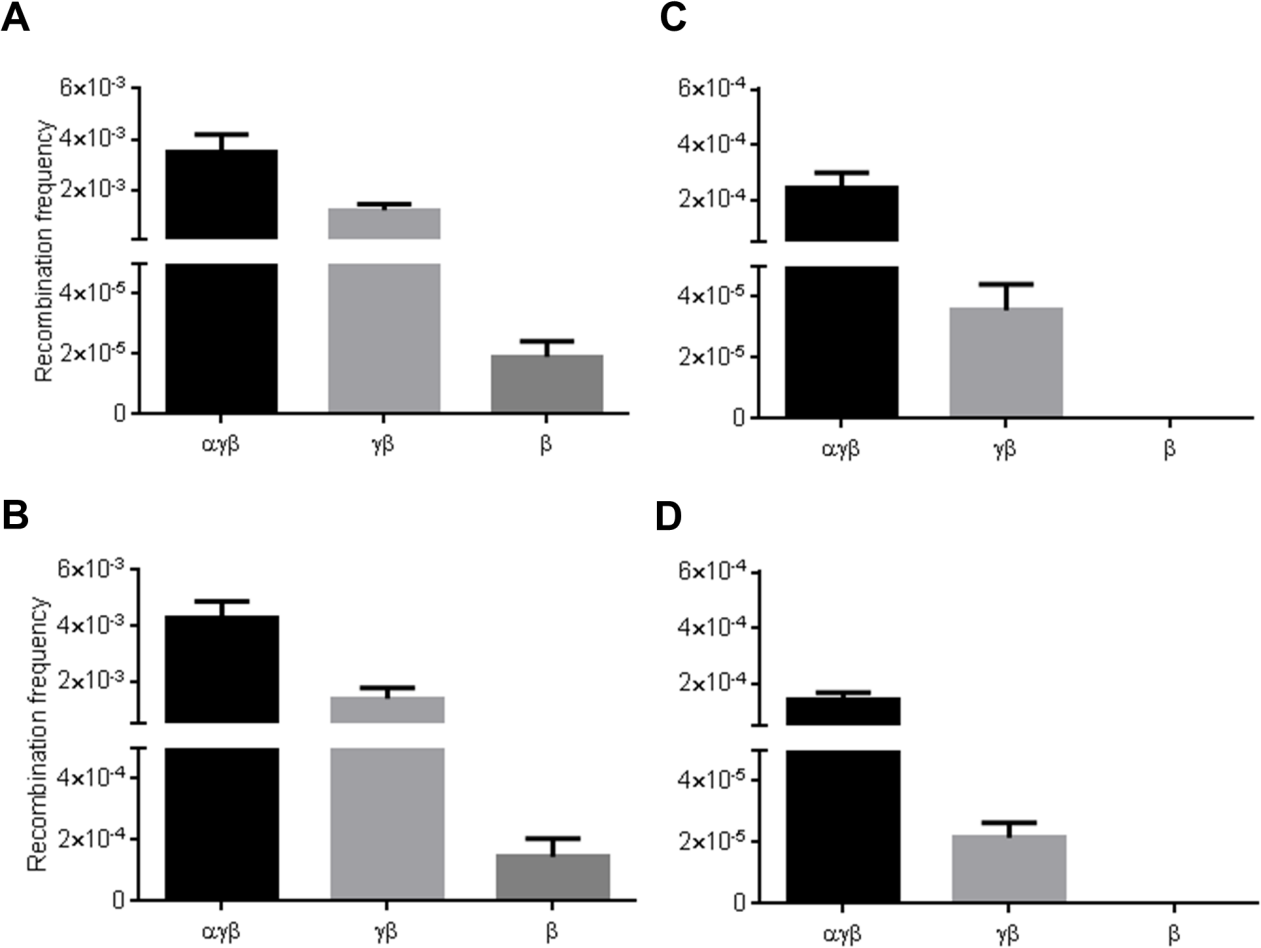
Requirement of different Red proteins for singleplex and multiplex recombination. (A) Insertion. A Gentamicin resistance cassette was inserted at a site of the *P2rx1* gene. (B) Gap repair. A p15A *zeo* subcloning plasmid was used to subclone a region of the *P2rx1* gene. (C) Multiplex insertion. Two different Gentamicin and Zeocin resistance cassettes were inserted at two different sites of the *P2rx1* gene. (D) SPI assay. The p15A *zeo* subcloning plasmid was used together with the Gentamicin resistance cassette. Recombination assays were performed using lagging strand protected cassettes and with expression of different Red proteins. Values represent average; error bars indicate standard error of mean (*n* = 3 for A, B and D, *n* = 4 for C).

The requirement of the different proteins of the Red system for singleplex and multiplex recombination was assessed by performing recombination assays with expression of different combinations of the Red proteins. Efficient insertion required Redγ protection of dsDNA and the lack of Redγ reduced recombination by over 60 fold in beta only expressing cells (Fig 5A). In contrast, gap repair was less sensitive and recombination was only 10 fold lower (Fig 5B). However, both multiplex insertion and SPI required Redγ since multiplex recombination was 183 fold and 399 fold less, respectively in beta cells (Fig 5C and 5D). While host exonucleases could substitute for Redα in singleplex insertion and gap repair assays albeit with slightly reduced efficiency (~3 fold), the absence of Redα resulted in a > 6-fold reduction in multiplex insertion and SPI (compare panels Fig 5A, 5C, 5B and 5D).

### Strategies to increase recombination efficiency

**Fig 6:**
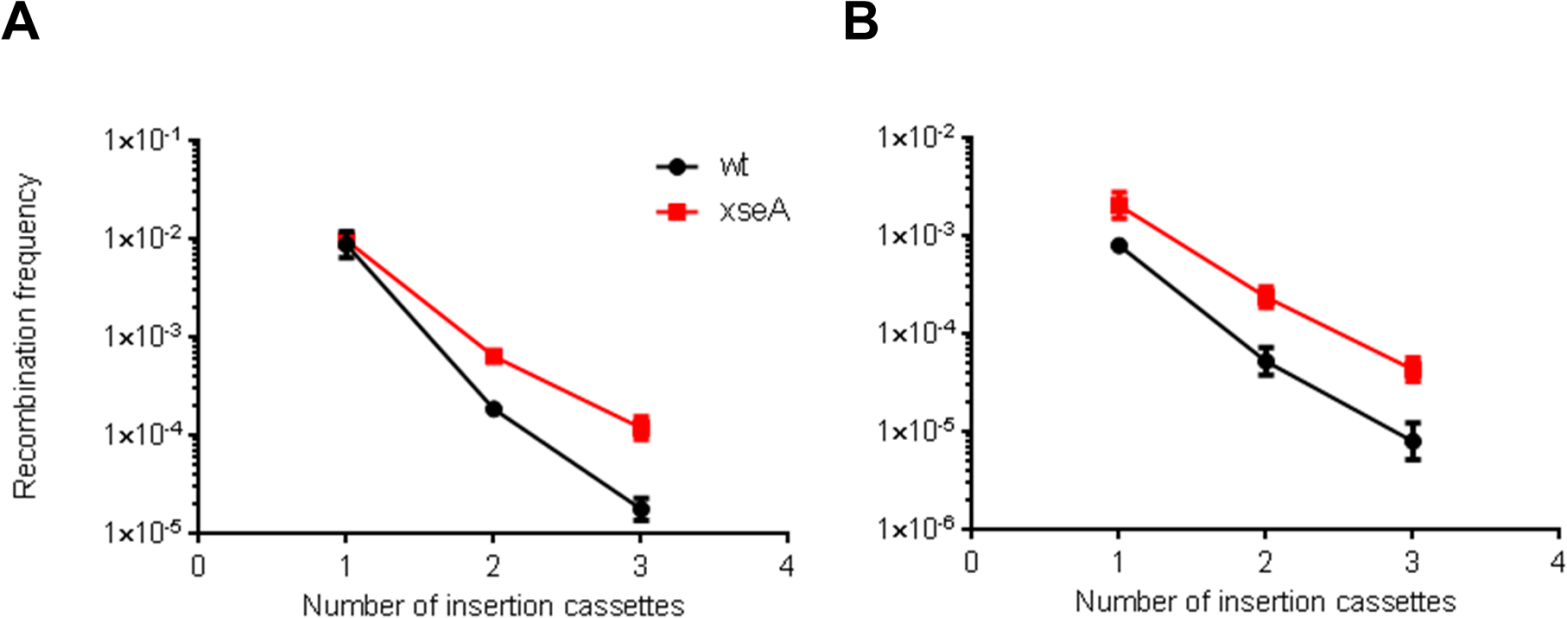
Multiplex recombination is increased in an Exo VII deletion strain. (A) Multiplex insertion. (B) SPI. Three different Gentamicin, Zeocin and Blasticidin lagging strand protected cassettes were inserted at three different sites of the *P2rx1* gene: 1 cassette, Gentamicin; 2 cassettes, Gentamicin and Zeocin; 3 cassettes, Gentamicin, Zeocin and Blasticidin. The p15A *dhfrII* lagging strand protected subcloning plasmid was used in the SPI assays. Data points represent averages; error bars indicate standard error of mean (*n* = 3 for A and B).

Recent work by Mosberg and colleagues [47] have identified Exo VII (*xseA*) as the primary nuclease responsible for degrading the ends of phosphorothioated dsDNA cassettes and oligos. An Exo VII knockout strain showed higher MAGE efficiency suggesting that a similar approach could increase multiplex recombination frequency of large DNA constructs. Multiplex insertion (Fig 6A) and SPI (Fig 6B) assays were performed in wt and Exo VII strains with different antibiotic cassettes targeting different sites of the *P2rx1* gene. The removal of Exo VII resulted in higher multiplex recombineering efficiencies particularly with increased cassette numbers (> 10 fold).

## Discussion

The development of recombineering tools in *E*. *coli* and other bacteria has enabled the introduction of a wide array of genetic modifications that were previously intractable to conventional methodologies [50–55]. In particular, the development of the highly multiplexed oligo recombineering technique of MAGE has enabled the construction of novel strains for use in bioprocess applications [34]. We have extended the repertoire of the recombineering toolbox with the development of multiplex recombineering using large DNA constructs. One useful application of this technique already is the rapid assembly of complex plasmid constructs like gene targeting vectors [45]. Multiplex recombineering could also be leveraged for the integration of whole genes and operons to construct microorganisms that contain novel biosynthetic pathways. To determine the key parameters that effect multiplex recombination efficiency, we have systematically compared singleplex and multiplex recombineering.

A key determinant of recombination is homology length. Biochemical and functional analysis of the mechanism of beta recombination have revealed a minimum requirement of 35 bp of sequence identity to the target region [4,15,20–22]. Recombination using shorter homologies involves a Red independent mechanism that is less efficient [56]. We found that both singleplex and multiplex recombination were most efficient with long homologies (> 120 bp). However, while insertion of one cassette required a minimum homology of 35 bp, insertion of two cassettes required 60 bp. Shorter homologies generated mixtures of targeted and non-targeted plasmids suggesting that higher recombination efficiency is required for multiplex recombination on the same DNA. Higher recombineering efficiency may also allow recombination with lower amounts of the DNA, which is expected in vivo due to the constraints of introducing sufficient quantities of the multiple DNA cassettes into the same cell and the degradation of the DNA by endogenous exonucleases [47]. Indeed, multiplex recombination was more reduced than singleplex recombination when lower amounts of the DNA cassettes were used in electroporation. Supporting this premise, phosphorothioate protection of DNA substantially increased multiplex recombination frequency.

One of the potential factors responsible for the high efficiency of multiplex recombineering is co-selection [40,49]. Indeed, multiplex recombination frequency was only ~10 fold reduced compared to singleplex recombination when selection was applied in both cases (see Fig 5). Consistent with this data, we observed a greater recovery of multiplex recombinants using co-selection than without. Co-selection has been previously used in enhanced MAGE termed CoS-MAGE [40] and a similar implementation could allow MAGE with large dsDNA cassettes lacking selection markers. Singleplex and multiplex recombination exhibited differential requirements for the Red proteins. Multiplex recombination required all three Red proteins for efficient recombination. The loss of exo-beta synergy [25,57] due to the lack of Redα had a greater impact on multiplex recombination than on singleplex recombination. These results support previous observations that other *E*. *coli* or phage encoded exonucleases cannot form a functional cognate pair with Redβ [20]. The sensitivity of multiplex recombination to template availability was again demonstrated with the lack of Redγ, which had a more detrimental effect on multiplex recombination than on singleplex recombination. Exploring strategies to increase the amount of DNA available in vivo using an ExoVII deletion strain greatly increased multiplex recombination. It is likely that optimizing DNA transformation and using the DnaG Q576A mutant strain that has been previously used in improved MAGE [58] could further improve multiplex cassette insertion. In conclusion, we have identified differential requirements to perform multiplex recombineering (summary in Table 1) that should help guide the application of Red recombination in various genetic engineering applications.

**Table 1:** Comparision of parameters affecting multiplex recombination of oligos and dsDNA cassettes.

Parameters^a^	ssDNA oligo	dsDNA cassettes
Minimum homology length^b^	15 bp	60 bp
Optimal homology length	35–45 bp	180 bp
Phosphorothioate modification	Yes	Yes
Replication fork target	Lagging strand	Lagging strand
Co-selection enhancement	Yes	Yes
Mismatch evasion required	Yes (*mutS*)	No
Secondary structure effect	Yes (ΔGss < − 12.5 kcal/mol)	No
Requirement of Redγα	No	Yes
Insertion size	30 bp	> 1 kb
Oligo or cassette size	70–90 bp	1–3 kb
Amount of each DNA cassette	0.5–1 μM	100–200 ng
Loci effect	Small	Large

^a^ The parameters are ranked in order of importance.

^b^ The homology length of each of the 5' and 3' ends.

## Materials and Methods

### Plasmids, strains and oligos

The RP24-360O20 BAC clone (*E*. *coli* genotype: F– *mcr*A Δ(*mrr*-*hsd*RMS-*mcr*BC) Φ80*lac*ZΔM15 Δ*lac*X74 *rec*A1 *end*A1 *ara*D139 Δ(*ara leu*) 7697 *gal*U *gal*K *rps*L *nup*G λ) containing the full-length mouse *P2rx1* gene was used in all the recombination assays. The insertion sites and the subcloning region is shown in S1 Fig. The *P2rx1* BAC was transformed with different pSC101^*ts*^ recombineering plasmids [8,59] and propagated at 30°C with Tetracycline selection. Desalted oligos were purchased from IDT or Invitrogen (S2 Table).

### Insertion cassettes and subcloning plasmids

The linear DNA cassettes contained homology regions flanking an antibiotic resistance gene and additionally for subcloning plasmids a replication origin. The homology regions were chosen to avoid repeat sequences. Insertion cassettes were cloned into R6Kγ plasmids using standard recombineering methods [31]. Subcloning plasmids were constructed from PCR generated fragments or synthetic gBLOCKS (IDT) using infusion cloning (Clontech). The subcloning plasmids were linearized at a unique restriction site between the homology regions prior to PCR. The insertion cassettes and subcloning plasmids were PCR amplified with modified primers using the KOD Hotstart DNA polymerase system (Merck Millipore). Briefly, PCR reactions were performed in a 50 μl total volume and contained 1X KOD polymerase buffer, 1.5 mM MgSO_4_, 200 μM of dNTPs, 200 nM of oligos, 1.5M Betaine, 1% DMSO, 1U KOD Hotstart DNA polymerase and 10–25 ng of the plasmid template. An initial hotstart step of 95°C for 2 mins was followed by 30 cycles of 92°C for 10 secs, 55°C for 30 secs, 72°C for 30 secs. The PCR products were analyzed by agarose gel electrophoresis and purified using the MinElute PCR purification kit (Qiagen). Different dilutions of the purified PCR products were quantified by agarose gel electrophoresis and comparison to a λ-HindIII digest (Invitrogen).

### Recombination assays

An overnight growth of the *P2rx1* BAC culture was diluted 50 fold in fresh LB medium, pH 8.0 (Lennox) containing selective antibiotics (10 ml per sample). The culture was grown shaking at 30°C to an OD_600_ of 0.3. Red proteins were induced with addition of L-Arabinose and/or L-Rhamnose to 0.2% final concentration and the culture was grown shaking at 37°C for a further 45 min. The cells were washed three times each with 1 ml of cold 10% glycerol and centrifugation at 17, 949 *g* for 20 secs at 4°C. The insertion cassettes and subcloning plasmids (600 ng each) were added to a single cell suspension and the cells were electroporated using a BioRad Gene Pulser system with a setting of 1.8 kv, 200 Ω and 25 μF. The cells were then immediately recovered in 950 μl of LB pH 8 and grown for 1 hr at 37°C. Dilutions of the recovered culture were made in 10 mM Tris, 10 mM MgS0_4_, 0.01% Gelatin (TMG) buffer pH 7.4 and the cells were plated on LB agar pH 8 plates containing antibiotics or lacking antibiotics to obtain the viable cell count. The following antibiotic concentrations were used: 100 μg ml^-1^Ampicillin, 40 μg ml^-1^Blasticidin, 12.5 μg ml^-1^ Chloramphenicol, 2 μg ml^-1^ Gentamicin, 15 μg ml^-1^ Kanamycin, 4 μg ml^-1^ Tetracycline, 10 μg ml^-1^ Trimethoprim, 5 μg ml^-1^ Zeocin. Liquid cultures contained similar antibiotic concentrations except 1 μg ml^-1^ Gentamicin.

### Analysis of recombinants

Single colonies were picked into 200 μl of LB+antibiotics in a 96-well plate and grown overnight at 37°C. To check correct cassette insertion and subcloning, PCR genotyping was performed using the ReddyMix PCR system (Thermo Scientific). PCR reactions in 20 μl contained 0.97X Reddymix DNA polymerase mastermix, 1 μM each of an insert specific oligo and a homology region flanking oligo and 2 μl of the saturated overnight culture. Thermal cycling was performed with an initial incubation at 95°C for 10 mins, followed by 35 cycles of 95°C for 10 secs, 55°C for 30 secs, 72°C for 30 secs, and a final extension step at 72°C for 10 mins. The PCR products were analysed by agarose gel electrophoresis on a 2% agarose gel.

Recombinants containing inserts were grown in 5 ml cultures and plasmid DNA was prepared using a BAC miniprep protocol [60] or the QIAprep Spin Miniprep kit (Qiagen). Long range PCR was performed with 2.5 μl of the BAC miniprep to test multiplex recombination on the same BAC DNA using the KOD Hostart DNA polymerase kit (Merck Millipore). The PCR conditions were identical as described earlier except PCR was performed in 25 μl, contained two homology region flanking oligos for each allele, and 35 cycles of PCR were performed. Multi-copy plasmids were analysed by RE digests. PCR products and RE digests were analysed on a 1% agarose gel. Gel images were inverted in Adobe Photoshop and were manipulated in Microsoft Powerpoint to increase brightness and contrast (brightness, -40%; contrast, 40%).

### Colony counts

The total number of recombinants were divided by the number of viable cells for each experiment and plotted as the recombination frequency. The gap repair frequency was corrected for background empty vectors using PCR genotyping as described in the figure legends. Mean and standard error of mean (s.e.m) were calculated from multiple independent experiments.

## Supporting Information

S1 Fig *P2rx1* insertion sites and subcloned region. The closed boxes represent exons (2–14) and the open box represents the 3’UTR region. Insertion sites are labeled A to D. The subcloned region spans a 12 kb segment of the *P2rx1* gene and the intergenic spacer between *P2rx1* and *Camkk* genes. Arrow indicates the direction of replication fork movement.
(TIF)

S2 Fig Effect of homology length on SPI using two different insertion cassettes. A SPI assay was performed at the *P2rx1* gene using a p15A *dhfrII* lagging strand protected subcloning plasmid containing 230 bp homology regions and a homology series (20 bp, 35 bp, 60 bp, 90 bp, 120 bp and 180 bp) of two different Gentamicin and Zeocin lagging strand protected cassettes both containing the same HL. Data points represent averages; error bars indicate standard error of mean (*n* = 3).
(TIF)

S3 Fig SPI assay using different homology combinations of subcloning plasmid and insertion cassette. SPI was performed at the *P2rx1* gene using lagging strand protected cassettes and plasmids in combination as shown in the table. Values represent averages; error bars indicate standard error of mean (*n* = 3).
(TIF)

S1 Table Recombination frequencies of the different assays. (DOCX)

S2 Table Insertion cassettes, subcloning plasmids and oligos used in this study. (DOCX)
