# Supplementary figures and images for: Differential Requirements of Singleplex and Multiplex Recombineering of Large DNA Constructs
Source: PLoS One. 2015 May 8;10(5):e0125533. doi: 10.1371/journal.pone.0125533 (PMC4425527; doi:10.1371/journal.pone.0125533)

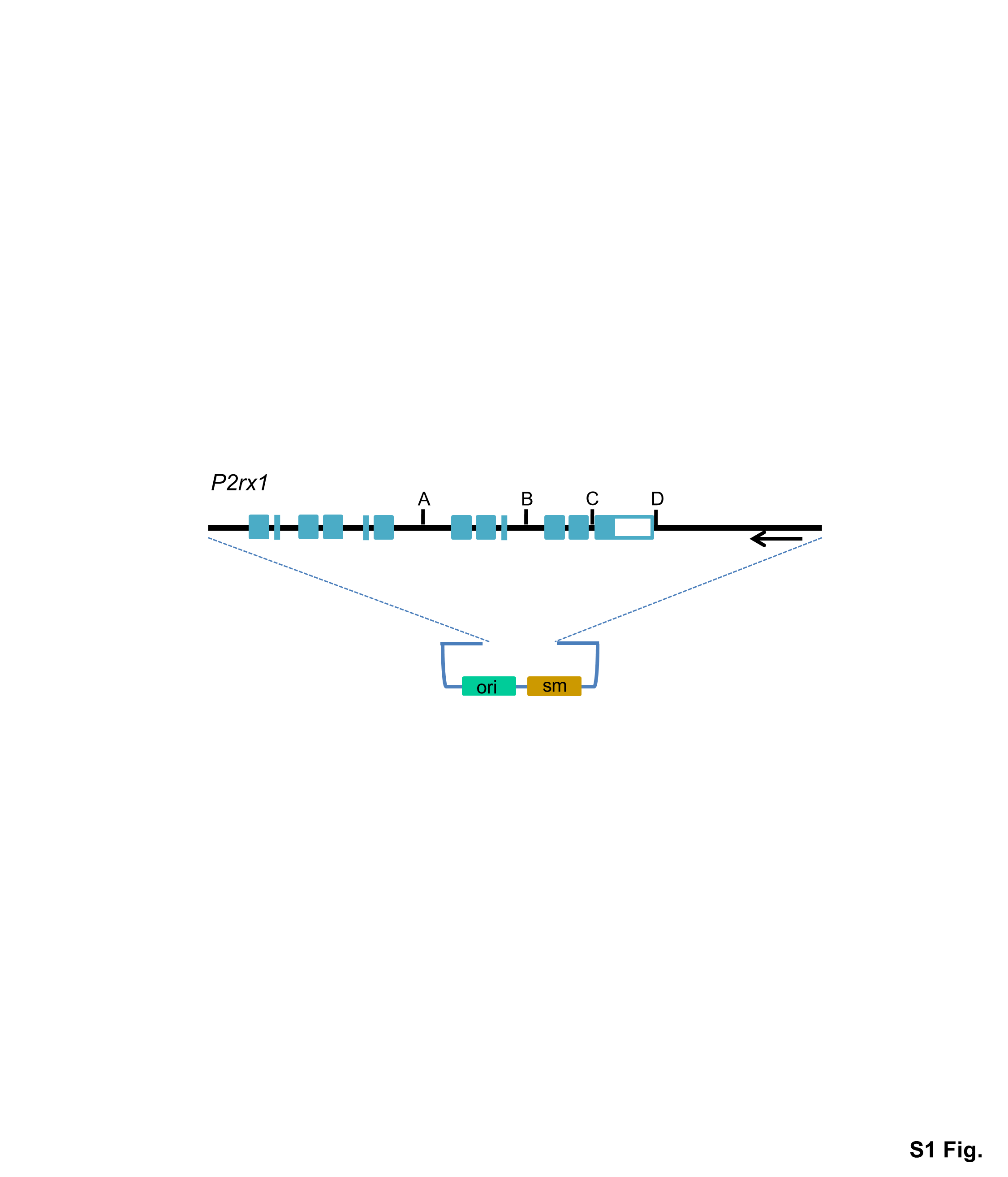

Supplement: S1 Fig — The closed boxes represent exons (2–14) and the open box represents the 3’UTR region. Insertion sites are labeled A to D. The subcloned region spans a 12 kb segment of the P2rx1 gene and the intergenic spacer between P2rx1 and Camkk genes. Arrow indicates the direction of replication fork movement. (TIF) [file pone.0125533.s001.tif]

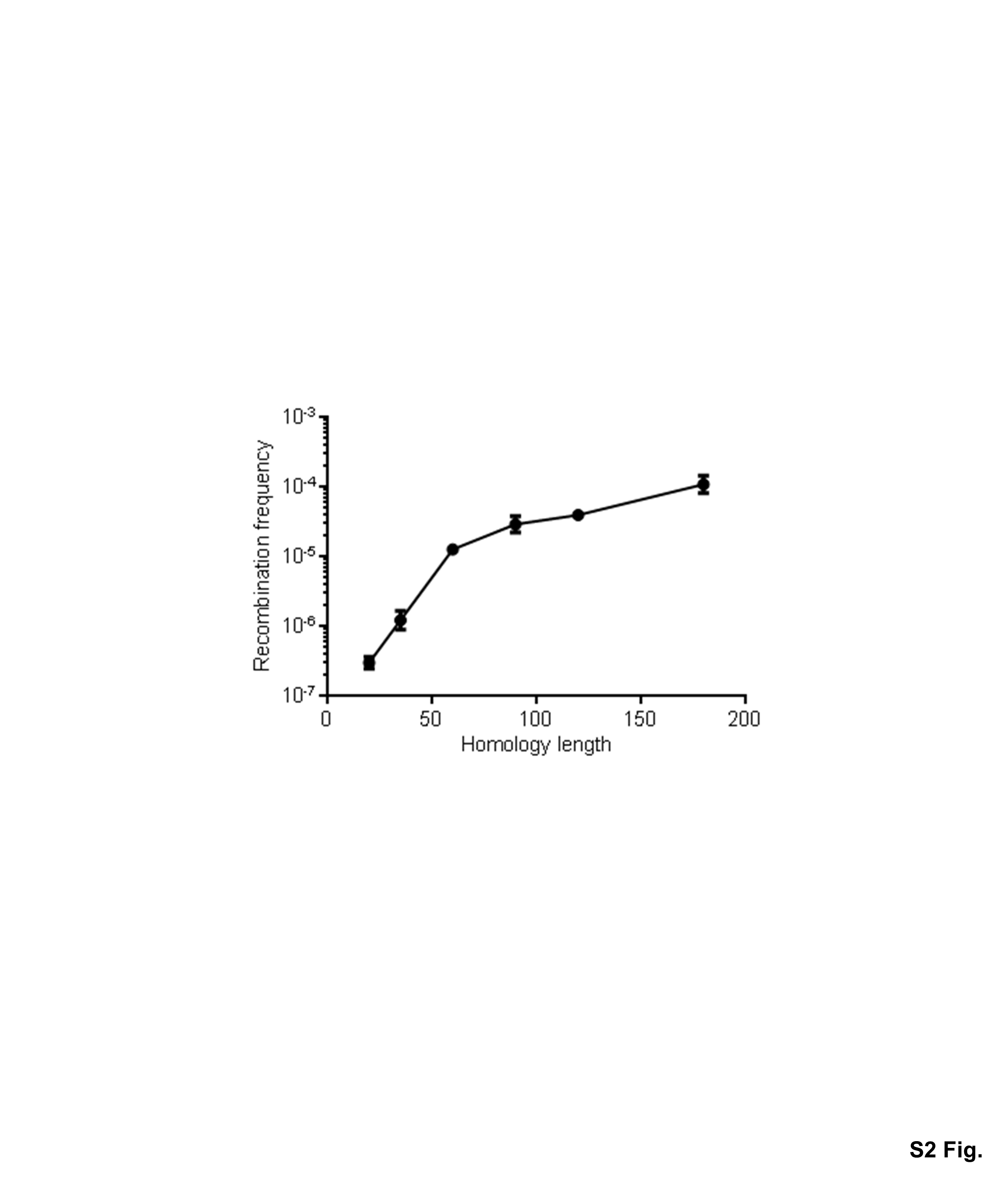

Supplement: S2 Fig — A SPI assay was performed at the P2rx1 gene using a p15A dhfrII lagging strand protected subcloning plasmid containing 230 bp homology regions and a homology series (20 bp, 35 bp, 60 bp, 90 bp, 120 bp and 180 bp) of two different Gentamicin and Zeocin lagging strand protected cassettes both containing the same HL. Data points represent averages; error bars indicate standard error of mean (n = 3). (TIF) [file pone.0125533.s002.tif]

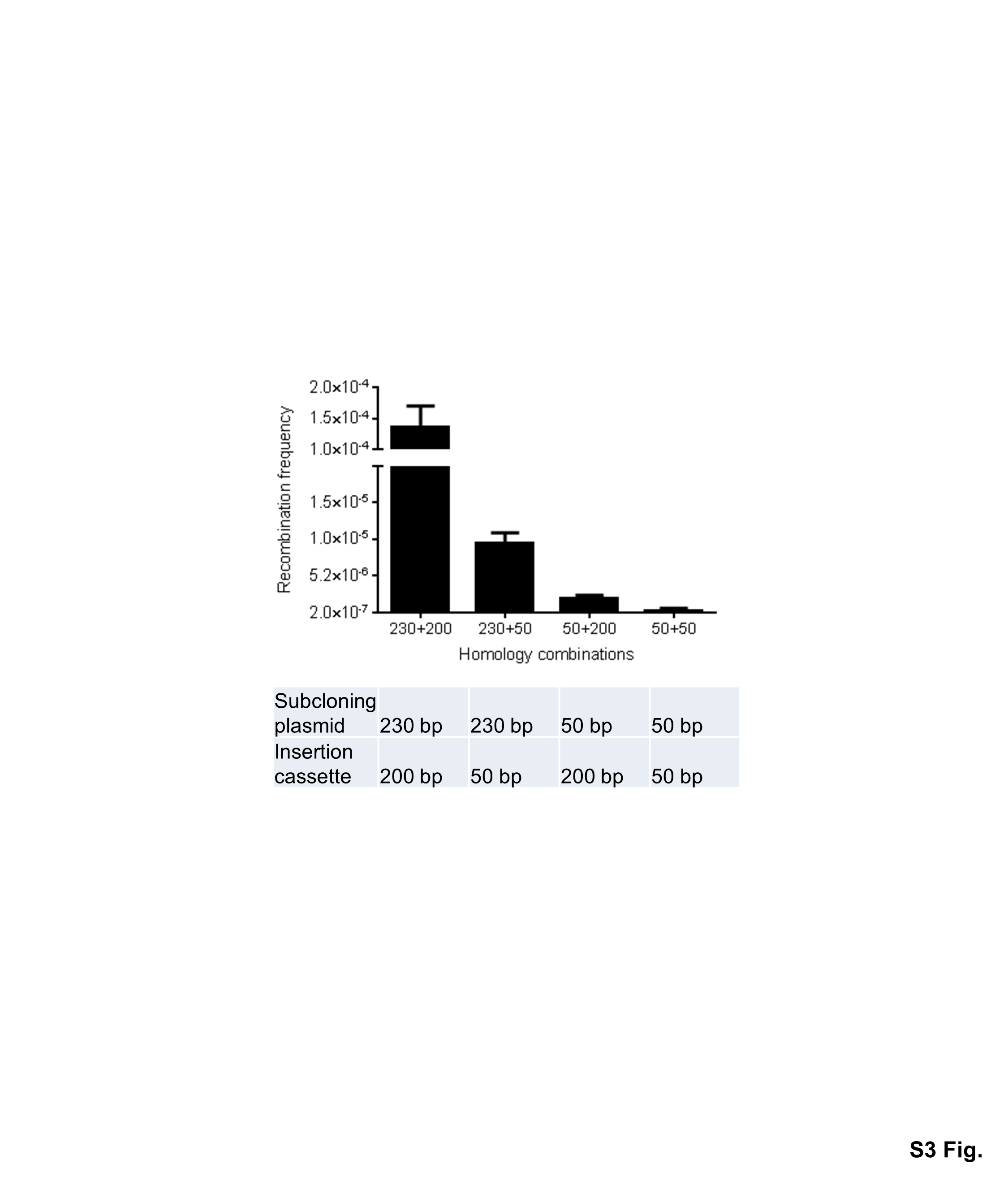

Supplement: S3 Fig — SPI was performed at the P2rx1 gene using lagging strand protected cassettes and plasmids in combination as shown in the table. Values represent averages; error bars indicate standard error of mean (n = 3). (TIF) [file pone.0125533.s003.tif]
