# Supplementary material for: Differential Requirements of Singleplex and Multiplex Recombineering of Large DNA Constructs
Source: PLoS One. 2015 May 8;10(5):e0125533. doi: 10.1371/journal.pone.0125533 (PMC4425527; doi:10.1371/journal.pone.0125533)
Supplement: S1 Table — (DOCX) [file pone.0125533.s004.docx]

**S1 Table Recombination frequencies of the different assays**

|  | | | | | | | | |
| --- | --- | --- | --- | --- | --- | --- | --- | --- |
| **Fig. 2A** |  |  |  |  |  |  |  |  |
|  | 20 | 35 | 60 | 90 | 120 | 180 |  |  |
| Average^a^ | 4.8E-05 | 7.2E-04 | 1.7E-03 | 4.7E-03 | 5.3E-03 | 0.009 |  |  |
| SEM^b^ | 2.5E-05 | 3.3E-04 | 1.2E-03 | 1.8E-03 | 1.7E-03 | 0.0020472 |  |  |
|  |  |  |  |  |  |  |  |  |
| **Fig. 2B** |  |  |  |  |  |  |  |  |
| Average | 0 | 5.6E-05 | 9.8E-04 | 2.5E-03 | 4.4E-03 | 7.9E-03 |  |  |
| SEM | 0 | 3.3E-05 | 4.4E-04 | 8.9E-04 | 2.2E-03 | 3.6E-03 |  |  |
|  |  |  |  |  |  |  |  |  |
| **Fig. 2C** |  |  |  |  |  |  |  |  |
| Average | 8.3E-07 | 8.0E-06 | 5.4E-05 | 1.4E-04 | 2.1E-04 | 4.1E-04 |  |  |
| SEM | 4.1E-07 | 5.7E-06 | 3.3E-05 | 9.9E-05 | 1.4E-04 | 1.8E-04 |  |  |
|  |  |  |  |  |  |  |  |  |
| **Fig. 2D** |  |  |  |  |  |  |  |  |
| Average | 4.1E-06 | 7.2E-05 | 2.1E-04 | 5.4E-04 | 6.1E-04 | 1.0E-03 |  |  |
| SEM | 2.1E-06 | 3.8E-06 | 7.9E-06 | 5.6E-05 | 4.9E-05 | 3.4E-05 |  |  |
|  |  |  |  |  |  |  |  |  |
| **Fig. 3A** |  |  |  |  |  |  |  |  |
|  | 0.1 ng | 1 ng | 10 ng | 100 ng |  |  |  |  |
| Average | 5.3E-06 | 4.5E-05 | 5.7E-04 | 4.2E-03 |  |  |  |  |
| SEM | 1.1E-06 | 9.9E-06 | 6.6E-05 | 1.3E-03 |  |  |  |  |
|  |  |  |  |  |  |  |  |  |
| **Fig. 3B** |  |  |  |  |  |  |  |  |
| Average | 0.0E+00 | 5.0E-08 | 9.9E-06 | 2.2E-04 |  |  |  |  |
| SEM | 0.0E+00 | 3.7E-09 | 1.9E-06 | 5.1E-05 |  |  |  |  |
|  |  |  |  |  |  |  |  |  |
| **Fig. 3C** |  |  |  |  |  |  |  |  |
|  | Un | Dual |  |  |  |  |  |  |
| Average | 2.0E-06 | 1.5E-05 |  |  |  |  |  |  |
| SEM | 1.3E-06 | 2.3E-06 |  |  |  |  |  |  |
|  |  |  |  |  |  |  |  |  |
| **Fig. 3D** |  |  |  |  |  |  |  |  |
| Average | 8.8E-07 | 1.3E-05 |  |  |  |  |  |  |
| SEM | 2.2E-08 | 5.8E-07 |  |  |  |  |  |  |
|  |  |  |  |  |  |  |  |  |
| **Fig. 4** |  |  |  |  |  |  |  |  |
|  | No selection | Co-selection |  |  |  |  |  |  |
| Average | 2.9E-07 | 5.3E-05 |  |  |  |  |  |  |
| SEM | 2.5E-07 | 2.4E-05 |  |  |  |  |  |  |
|  |  |  |  |  |  |  |  |  |
| **Fig. 5A** |  |  |  |  |  |  |  |  |
|  | γβα | γβ | β |  |  |  |  |  |
| Average | 3.5E-03 | 1.2E-03 | 1.9E-05 |  |  |  |  |  |
| SEM | 2.0E-03 | 6.9E-04 | 1.1E-05 |  |  |  |  |  |
|  |  |  |  |  |  |  |  |  |
| **Fig. 5B** |  |  |  |  |  |  |  |  |
| Average | 4.3E-03 | 1.4E-03 | 1.4E-04 |  |  |  |  |  |
| SEM | 2.5E-03 | 8.0E-04 | 8.3E-05 |  |  |  |  |  |
|  |  |  |  |  |  |  |  |  |
| **Fig. 5C** |  |  |  |  |  |  |  |  |
| Average | 3.4E-03 | 1.1E-03 | 1.1E-04 |  |  |  |  |  |
| SEM | 1.2E-04 | 1.8E-05 | 9.8E-08 |  |  |  |  |  |
|  |  |  |  |  |  |  |  |  |
| **Fig. 5D** |  |  |  |  |  |  |  |  |
| Average | 1.4E-04 | 2.2E-05 | 5.4E-08 |  |  |  |  |  |
| SEM | 8.1E-05 | 1.2E-05 | 3.1E-08 |  |  |  |  |  |
|  |  |  |  |  |  |  |  |  |
| **Fig. 6A** |  |  |  |  |  |  |  |  |
|  | wt | wt | wt | *xseA* | *xseA* | *xseA* |  |  |
|  | 1 | 2 | 3 | 1 | 2 | 3 |  |  |
| Average | 8.7E-03 | 1.8E-04 | 1.8E-05 | 9.6E-03 | 6.3E-04 | 1.2E-04 |  |  |
| SEM | 3.1E-03 | 3.5E-05 | 5.4E-06 | 8.8E-04 | 1.1E-04 | 3.7E-05 |  |  |
|  |  |  |  |  |  |  |  |  |
| **Fig. 6B** |  |  |  |  |  |  |  |  |
| Average | 7.9E-04 | 5.2E-05 | 7.9E-06 | 2.0E-03 | 2.4E-04 | 4.3E-05 |  |  |
| SEM | 1.5E-04 | 2.0E-05 | 4.3E-06 | 7.3E-04 | 6.5E-05 | 1.4E-05 |  |  |
|  |  |  |  |  |  |  |  |  |
| **Fig. S3** |  |  |  |  |  |  |  |  |
|  | 20 | 35 | 60 | 90 | 120 | 180 |  |  |
| Average | 3.0E-07 | 1.2E-06 | 1.3E-05 | 2.9E-05 | 3.9E-05 | 1.1E-04 |  |  |
| SEM | 6.2E-08 | 4.5E-07 | 1.2E-06 | 8.8E-06 | 7.2E-06 | 3.5E-05 |  |  |
|  |  |  |  |  |  |  |  |  |
| **Fig. S4** |  |  |  |  |  |  |  |  |
|  | 230+200 | 230+50 | 50+200 | 50+50 |  |  |  |  |
| Average | 1.4E-04 | 9.6E-06 | 2.1E-06 | 4.5E-07 |  |  |  |  |
| SEM | 3.3E-05 | 1.4E-06 | 4.0E-07 | 3.1E-07 |  |  |  |  |

^a^Recombination frequency was determined by dividing the total number of antibiotic resistant colonies by the total number of colonies surviving after electroporation. Gap repair values were corrected for aberrant recombinants. Average recombination frequency was determined from multiple independent experiments (see figure legends for the number of replicates).

^b^standard error of mean
