# Supplementary material for: Differential Requirements of Singleplex and Multiplex Recombineering of Large DNA Constructs
Source: PLoS One. 2015 May 8;10(5):e0125533. doi: 10.1371/journal.pone.0125533 (PMC4425527; doi:10.1371/journal.pone.0125533)
Supplement: S2 Table — (DOCX) [file pone.0125533.s005.docx]

**S2 Table Insertion cassettes, subcloning plasmids and oligos used in this study.**

|  |
| --- |

Insertion cassettes:

Homology regions

EM7 promoter

Antibiotic resistance marker

*P2rx1* site A Blasticidin cassette:

CACCTGCTAGTTAGCATTGCAGCTCTGCCCTGCTGGGACAATCCTATACCCTGTTCTTGCTGGATCCTTCTTGTGCCTGCTTCTGTAGCTGCCTATGTGGGAATGGTAACCAGTCCTTTTTTTCTTTTTTTATTTCCTGTGAGATCACCAACACACCCCACTTAGGACACTTGTGTATGGCAGAGGCCTGCAGATGTTTGCTTTCACGACCCACATGGAGGTGGAAGCAGCCAGACAGTGGTTAA

GTTGACAATTAATCATCGGCATAGTATATCGGCATAGTATAATACGACAAGGTGAGGAACTAAACC

ATGGCCAAGCCTTTGTCTCAAGAAGAATCCACCCTCATTGAAAGAGCAACGGCTACAATCAACAGCATCCCCATCTCTGAAGACTACAGCGTCGCCAGCGCAGCTCTCTCTAGCGACGGCCGCATCTTCACTGGTGTCAATGTATATCATTTTACTGGGGGACCTTGTGCAGAACTCGTGGTGCTGGGCACTGCTGCTGCTGCGGCAGCTGGCAACCTGACTTGTATCGTCGCGATCGGAAATGAGAACAGGGGCATCTTGAGCCCCTGCGGACGGTGTCGACAGGTGCTTCTCGATCTGCATCCTGGGATCAAAGCGATAGTGAAGGACAGTGATGGACAGCCGACGGCAGTTGGGATTCGTGAATTGCTGCCCTCTGGTTATGTGTGGGAGGGCTAA

CTGTGGATGTTCAAGCATCAGTTTGCCTGATCCTATAGGGCTGTCATGCTTTTACAATCTGCCTGTTTCTAGAACCCACGCCTGCCCTCACCCACTGAGAGGTCTGGATCTCTGTTCCCACCTCCCCACTGAGTGGCTCAGCTCCCTGCCCACTTCTCTAATCACCTCCCCACTCTTGGCCCGAGCCACCCACCTGCCCTGTTCCTTGCCTCCCTTCACTTTCTGAAGTATTTTCTGCTCAGACTTCACAGG

*P2rx1* site B Zeocin cassette:

GATCTGCTTTTAATGACCATGGGTATAGGTGATGACCCTGGCCAGGCTTTGAGCCACTGCCATGGCTCTCCTACCATCTCCTTGGGTCGAGGGGAACAAGGTTAGAGTCAAAGCTCAGGTTTACTGACCTGGAGCTGGCTCTCAGGAGAGCCACAGCCCTGGATCACTGGTAGCAGGAATAGTGTGGAATTGTAGGAGGCAGGTTTGACTTTCC

GTTGACAATTAATCATCGGCATAGTATATCGGCATAGTATAATACGACAAGGTGAGGAACTAAACC

ATGGCCAAGTTGACCAGTGCCGTTCCGGTGCTCACCGCGCGCGACGTCGCCGGAGCGGTCGAGTTCTGGACCGACCGGCTCGGGTTCTCCCGGGACTTCGTGGAGGACGACTTCGCCGGTGTGGTCCGGGACGACGTGACCCTGTTCATCAGCGCGGTCCAGGACCAGGTGGTGCCGGACAACACCCTGGCCTGGGTGTGGGTGCGCGGCCTGGACGAGCTGTACGCCGAGTGGTCGGAGGTCGTGTCCACGAACTTCCGGGACGCCTCCGGGCCGGCCATGACCGAGATCGGCGAGCAGCCGTGGGGGCGGGAGTTCGCCCTGCGCGACCCGGCCGGCAACTGCGTGCACTTCGTGGCCGAGGAGCAGGACTGA

CTAGCTGGGCCCCTTCTGTTGACCTGGCTTTCCCCCTGGGCCCTGCCCAACCAGGCTGGGAAGTTTGACATCATCCCTACTATGACTACCATCGGCTCTGGGATTGGCATCTTTGGAGTGGTAAGTACTGGGGATACATGTTTGCTGTCTGGAGTTTGGCTGCTACTCTTCTACACACAGTAGCAGAACCCCAAAATGTGACCATAGCTACCTTTCATGAATGAAACAGC

*P2rx1* site D Gentamicin cassette:

AAATGGCTGTTTTTTGCTTTCCTTTCAAGCCTGTCTGAAGTTCAGTGTGCTTGCCTGTGAGATGCACAGCTGCGCTGAACAGCTGAGATCCCTGTGAGAAGGCTGGTGAGGGGTGCCTGGGCCAAGATGAGCCAGTGAGCCTACAGCAGTCCCTGAGCTCTGTCTCTGCACAGGACAGGGCACCGGTGCTCTTCCCAATAGCAAGGAACAAGTTCTTCTGGC

GTTGACAATTAATCATCGGCATAGTATATCGGCATAGTATAATACGACAAGGTGAGGAACTAAACC

ATGTTACGCAGCAGCAACGATGTTACGCAGCAGGGCAGTCGCCCTAAAACAAAGTTAGGTGGCTCAAGTATGGGCATCATTCGCACATGTAGGCTCGGCCCTGACCAAGTCAAATCCATGCGGGCTGCTCTTGATCTTTTCGGTCGTGAGTTCGGAGACGTAGCCACCTACTCCCAACATCAGCCGGACTCCGATTACCTCGGGAACTTGCTCCGTAGTAAGACATTCATCGCGCTTGCTGCCTTCGACCAAGAAGCGGTTGTTGGCGCTCTCGCGGCTTACGTTCTGCCCAGGTTTGAGCAGCCGCGTAGTGAGATCTATATCTATGATCTCGCAGTCTCCGGCGAGCACCGGAGGCAGGGCATTGCCACCGCGCTCATCAATCTCCTCAAGCATGAGGCCAACGCGCTTGGTGCTTATGTGATCTACGTGCAAGCAGATTACGGTGACGATCCCGCAGTGGCTCTCTATACAAAGTTGGGCATACGGGAAGAAGTGATGCACTTTGATATCGACCCAAGTACCGCCACCTAA

TGTGGGGCAGTTGAGCACACCAGAAGCAGCCTTGAGGGAGCATGGTTGAGGGAGGGGCTTGTTGTCCCAGGGATCTGGGGACACTTCCACGGACATGAGGCTTGCAGCTGGAGAGTAGAGAAGGCCACATAGGCACACCCTCCAGCCTGGGCTCACCTCCAACCAGGGCTGCTAAGTACCTCCACCCCTGCTCTGTCTAGACCA

Subcloning plasmid:

Zeocin or Trimethoprim resistance cassette

p15A origin

*P2rx1* homology regions

NotI site

TCAGTCCTGCTCCTCGGCCACGAAGTGCACGCAGTTGCCGGCCGGGTCGCGCAGGGCGAACTCCCGCCCCCACGGCTGCTCGCCGATCTCGGTCATGGCCGGCCCGGAGGCGTCCCGGAAGTTCGTGGACACGACCTCCGACCACTCGGCGTACAGCTCGTCCAGGCCGCGCACCCACACCCAGGCCAGGGTGTTGTCCGGCACCACCTGGTCCTGGACCGCGCTGATGAACAGGGTCACGTCGTCCCGGACCACACCGGCGAAGTCGTCCTCCACGAAGTCCCGGGAGAACCCGAGCCGGTCGGTCCAGAACTCGACCGCTCCGGCGACGTCGCGCGCGGTGAGCACCGGAACGGCACTGGTCAACTTGGCCATGATTGTCCTCCTGGTTTAGTTCCTCACCTTGTCGTATTATACTATGCCGATATACTATGCCGATGATTAATTGTCAAC

Or

GGACTAGTGCAAAAAACCCCTCAAGACCCGTTTAGAGGCCCCAAGGGGTTATGCTAGTTTGAACTCAGTTGATGCGTTCAAGCGCCGCAACAGGATAAATCTGTACTGAGCCTGGGTGAGCCTCAGACTCGACGGCGTAGCCTTCGGGGGTCAAATTTGTGCAGTACCACCCGACAATCTGACCTTGCCAGGCGGCGCCGGATTTCTTGCGCACGCGATCTCCCATACCAAACGTGGCGTTCGATGGGAATACAAAATTGCCAGCAACTGGATTACTGACTTCATTGCTACTTCGTTCCATACTTTTCCTTTTTCAATATTATTGAAGCATTTATCAGGGTTATTGTCTCATCTGTCAAGATCTTC

CGGTGACCCGGGTCTTAATTAATAAGATGATCTTCTTGAGATCGTTTTGGTCTGCGCGTAATCTCTTGCTCTGAAAACGAAAAAACCGCCTTGCAGGGCGGTTTTTCGAAGGTTCTCTGAGCTACCAACTCTTTGAACCGAGGTAACTGGCTTGGAGGAGCGCAGTCACCAAAACTTGTCCTTTCAGTTTAGCCTTAACCGGCGCATGACTTCAAGACTAACTCCTCTAAATCAATTACCAGTGGCTGCTGCCAGTGGTGCTTTTGCATGTCTTTCCGGGTTGGACTCAAGACGATAGTTACCGGATAAGGCGCAGCGGTCGGACTGAACGGGGGGTTCGTGCATACAGTCCAGCTTGGAGCGAACTGCCTACCCGGAACTGAGTGTCAGGCGTGGAATGAGACAAACGCGGCCATAACAGCGGAATGACACCGGTAAACCGAAAGGCAGGAACAGGAGAGCGCACGAGGGAGCCGCCAGGGGGAAACGCCTGGTATCTTTATAGTCCTGTCGGGTTTCGCCACCACTGATTTGAGCGTCAGATTTCGTGATGCTTGTCAGGGGGGCGGAGCCTATGGAAAAACGGCTTTGCCGCGGCCCTCTCACTTCCCTGTTAAGTATCTTCCTGGCATCTTCCAGGAAATCTCCGCCCCGTTCGTAAGCCATTTCCGCTCGCCGCAGTCGAACGACCGAGCGTAGCGAGTCAGTGAGCGAGGAAGCGGAATATATCCTGTATCACATATTCTGCTGACGCACCGGTGCAGCCTTTTTTCTCCTGCCACATGAAGCACTTCACTGACACCCTCATCAGTGCCAACATAGTAAGCCAGTATACACTCCGCTAGCGCTTAATTAACCTGCAGG

AGGATACCAGACCTCAAGTGGCCTTATCAGCAGTGTGTCAGTGAAACTCAAGGGCTTGGCTGTGACCCAGCTCCAGGGCCTGGGACCCCAGGTCTGGGACGTGGCTGACTATGTCTTCCCAGCACATGTAAGCGGCACCATCCTTCCTCCCATAAGCCCTGGCTTAAGGCTCTTTGAGGGACCAGTCCAGCTTCCTGCTCTCTGGGCCTCAGTCTGCCTTACTGTGCGAC

GCGGCCGC

AGGACTGTGTCCCAGTGAGCTCCTCTCTTTGTCTTGGCCAGGTTCAGGGTGTGAGAGAAGCGCACAGCTGTGTCCTTGAAAATCCCAGGGAAAAGGAAGAGGCTGGGTCTTCTGCGCATCTCTACCTCTTCCATCCAACATACGCTGTGGGTCAGACTGTTCTAGACCCTACTACCTGCTCCTTGGTCCCCCACGCCCAGTCCTCAGACCAGGACTGGGGCTTATTTTAAACCTTATCCAAGTGAATTGC

Oligos:

Fig. 1: *P2rx1* site D Gentamicin cassette

HA G 20 F: A*A*GGAACAAGTTCTTCTGGC

HA G 20 R: /5PHOS/GTGTGCTCAACTGCCCCACA

HA G 35 F: G*C*TCTTCCCAATAGCAAGGA

HA G 35 R: /5PHOS/TCAAGGCTGCTTCTGGTGTG

HA G 60 F: T*C*TCTGCACAGGACAGGGCA

HA G 60 R: /5PHOS/AAGCCCCTCCCTCAACCATG

HA G 120 F: C*T*GGTGAGGGGTGCCTGGGC

HA G 120 R: /5PHOS/CTCTACTCTCCAGCTGCAAG

HA G 180 F: C*A*GTGTGCTTGCCTGTGAGA

HA G 180 R: /5PHOS/GGTACTTAGCAGCCCTGGTT

Fig. 1B: p15A *P2rx1* subcloning plasmids

p15A 20 F: A*C*CTTATCCAAGTGAATTGC

p15A 20 R: /5Phos/cacttgaggtctggtatccT

p15A 35 F: T*G*GGGCTTATTTTAAACCTT

p15A 35 R: /5Phos/CACTGCTGATAAGGCCACTT

p15A 60 F: C*C*ACGCCCAGTCCTCAGACCAGGACTGGGGCTTATTTTAAACCTTATCCAAGTGAATTGCTCAGTCCTGCTCCTCGGCCA

p15A 60 R: /5PHOS/GCCAAGCCCTTGAGTTTCACTGACACACTGCTGATAAGGCCACTTGAGGTCTGGTATCCTCCTGCAGGTTAATTAAGCGC

p15A 90F: T*C*TAGACCCTACTACCTGCT

p15A 90 R: /5PHOS/TGGGGTCCCAGGCCCTGGAG

p15A 120 F: C*C*ATCCAACATACGCTGTGG

p15A 120 R: /5PHOS/GGGAAGACATAGTCAGCCAC

p15A 180 F: T*G*TCCTTGAAAATCCCAGG

p15A 180 R: /5PHOS/CCCTCAAAGAGCCTTAAGCCAG

Fig. 1C: *P2rx1* site B Zeocin cassette

HA Z 20 F: G*G*AGGCAGGTTTGACTTTCC

HA Z 20 R: /5PHOS/AACAGAAGGGGCCCAGCTAG

HA Z 35 F: T*A*GTGTGGAAT TGTA GGAGG

HA Z 35 R: /5PHOS/GGGGAAAGCCAGGTCAACAG

HA Z 60 F: A*G*CCCTGGATCACTGGTAGC

HA Z 60 R: /5PHOS/CCCAGCCTGGTTGGGCAGGGCCCAGGGGGA

HA Z 90 F: T*G*ACCTGGAGCTGGCTCTCA

HA Z 90 R: /5PHOS/GGTAGTCATAGTAGGGATGA

HA Z 120 F: A*A*CAAGGTTAGAGTCAAAGC

HA Z 120 R: /5PHOS/CACTCCAAAGATGCCAATCC

HA Z 180 F: A*C*CCTGGCCAGGCTTTGAGC

HA Z 180 R: /5PHOS/CTGTGTGTAGAAGAGTAGCA

Fig. 1D: p15A *P2rx1* subcloning plasmid

A*G*GACTGTGTCCCAGTGAGC

/5PHOS/GTCGCACAGTAAGGCAGACT

Figs. 2, 3, 4 and 5:

*P2rx1* site A Bsd F: C*A*CCTGCTAGTTAGCATTGC

*P2rx1* site A Bsd R: /5PHOS/CCTGTGAAGTCTGAGCAGAA

*P2rx1* site B Zeo F: G*A*TCTGCTTTTAATGACCATGGGTA

*P2rx1* site B Zeo R: /5PHOS/GCTGTTTCATTCATGAAAGGTAGC

*P2rx1* site D Genta F: A*A*ATGGCTGTTTTTTGCTTTCC

*P2rx1* site D Genta R: /5PHOS/TGGTCTAGACAGAGCAGGGGT

Fig. 6:

*P2rx1* site A Blasticidin cassette

*P2rx1* site A Bsd F: C*A*CCTGCTAGTTAGCATTGC

*P2rx1* site A Bsd R: /5PHOS/CCTGTGAAGTCTGAGCAGAA

*P2rx1* site B Zeocin:

HA Z 180 F: A*C*CCTGGCCAGGCTTTGAGC

HA Z 180 R: /5PHOS/CTGTGTGTAGAAGAGTAGCA

*P2rx1* site D Gentamicin:

HA G 180 F: C*A*GTGTGCTTGCCTGTGAGA

HA G 180 R: /5PHOS/GGTACTTAGCAGCCCTGGTT

Asterisk denotes phosphorothioate bonds

Colony PCR genotyping oligos:

Site A: 415 bp

*P2rx1* site A F: CCAATGTGGGAGCCATGAGGA

*P2rx1* *bsd* R: GGGATGCTGTTGATTGTAGCCGT

Site B: 399 bp

*P2rx1* site B F: GGCACCTTCCTGCTTTGGATGT

*P2rx1* *zeo* R: CCGGAACGGCACTGGTCAA

Site D: 581 bp

*P2rx1* site D F: TGAGGGCTGAGGAGGCAAGG

*P2rx1* *genta* R: GAAGGCAGCAAGCGCGATGA

*P2rx1* subcloned insert: 353 bp^a^

p15A *zeo P2rx1* 3' F: TGTCAGGGAAGGGGTGTGTGT

p15A *zeo P2rx1* 3' R: ACTTCGTGGCCGAGGAGCAG

^a^Aberrant recombinants produced 2 bands with this PCR assay. RE digests and DNA sequencing confirmed that they lacked the full length *P2rx1* insert.
